# Multimodal image characterization of paravenous atrophy

**DOI:** 10.3205/oc000116

**Published:** 2019-07-16

**Authors:** Carmen Alba-Linero, Glenda Espinosa Barberi, Victor Llorens, María Socorro Alforja Castella, Alfredo Adán

**Affiliations:** 1 Clinic Institute of Ophthalmology, Clinic Hospital of Barcelona, Spain; 2 Ophthalmology Department, Doctor Negrín University Hospital, Las Palmas de Gran Canaria, Spain

**Keywords:** paravenous atrophy, multimodal image, autofluorescence, retinitis pigmentosa-like, optical coherence tomography angiography

## Abstract

**Objective:** The objective is to describe a clinical case of paravenous atrophy using a multimodal image.

**Methods:** A 48-year-old man was visited and followed in the ophthalmology department of Hospital Clinic (Barcelona). Visual acuity, slit-lamp exam, retinography, autofluorescence, visual field, optical coherence tomography, and electrophysiology test were performed.

**Results:** The patient had a history of Behcet’s disease. Autofluorescence revealed hypoautofluorescence at the paravenous region, th visual field was also altered and electrophysiologic test were reduced.

**Conclusion:** Paravenous atrophy is a rare entity not well described currently. Patients are frequently misdiagnosed. Multimodal image could help to characterize this condition properly and improve the management.

## Introduction

Pigmented paravenous retinochoroidal atrophy (PPRCA), described as retino-choroiditis radiata in 1937 [1], is an uncommon disease with unknown etiology characterized by bilateral retinal pigment epithelium (RPE) and choroidal atrophy along retinal veins, generally without macular involvement. The main physiopathologic theory supports that choroidal thinning precedes development of the RPE atrophy [2]. PPRCA is commonly bilateral and symmetric, but appears to have variable expressivity and a spectrum of mild to severe ophthalmoscopic change [3]. 

Diagnosis is often fortuitously made during a routine exam and is based on a typical and characteristic fundus appearance. The principal differential diagnosis has to be done with typical retinitis pigmentosa (RP) [4].

Non-invasive imaging techniques including retinography, fundus autofluorescence (FAF), swept source optical coherence tomography (SS-OCT), and OCT angiography (Angio-OCT) are now available to the study of retinal dystrophies like this. We present a clinical case of PPRCA in association with Behcet's disease through multimodal study.

## Case description

A 48-year-old man of Caucasian origin and ex-smoker came to our ophthalmology department with visual and campimetric worsening in both eyes (OU) since the last two months. He had a history of Behcet’s disease with severe ocular involvement (retinochoroidal) and bipolar aphtosis, previously treated with immunosuppressants and biologic therapy: adalimumab (ADA), infliximab, and azatriopine (AZA). At that time he was stable (no signs of activity) and following treatment with ADA 40 mg/month. 

**Figure 1:**
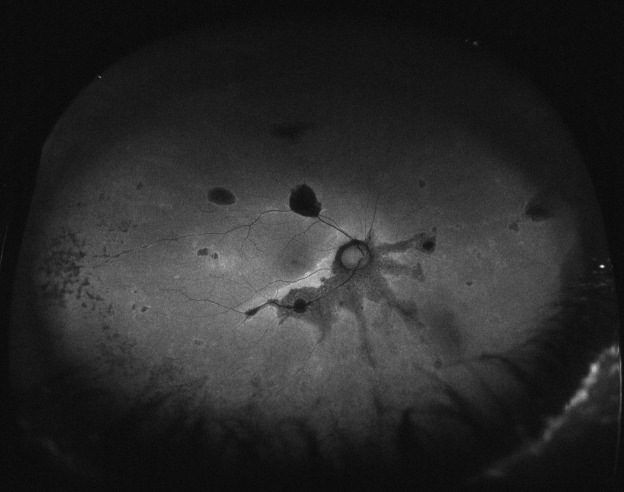
Autofluorescence image of the right eye showing hypoautofluorescence at the paravenous zone

**Figure 2:**
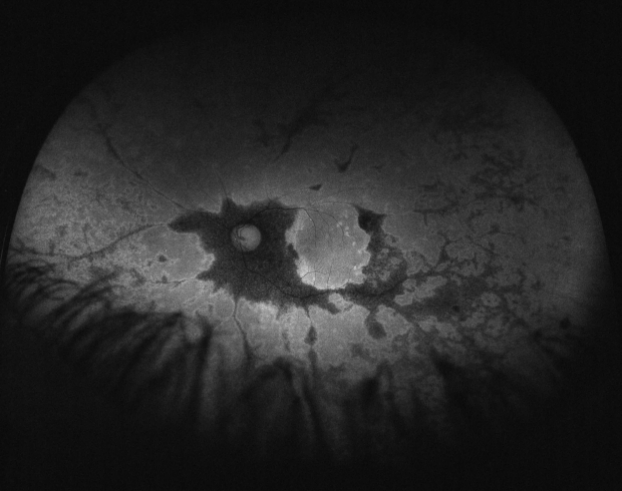
Autofluorescence image of the left eye demostrating hypoautofluorescence at the paravenous zone

**Figure 3:**
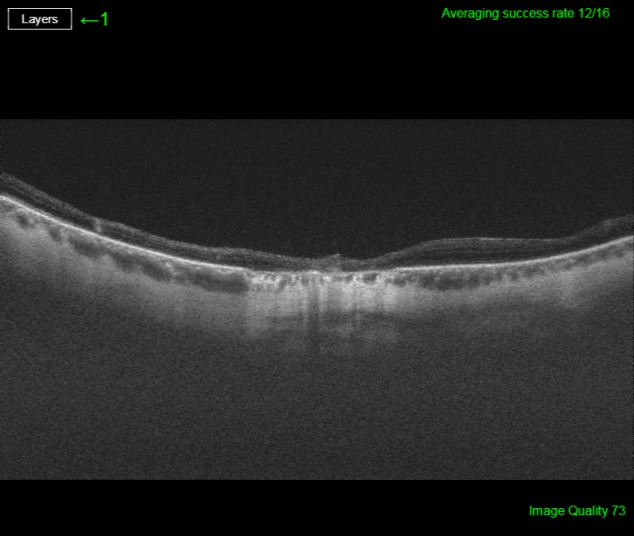
SS-OCT image of the right eye reveals external retina altered with an increase of choroidal signal due to retinal pigment epithelium atrophy.

**Figure 4:**
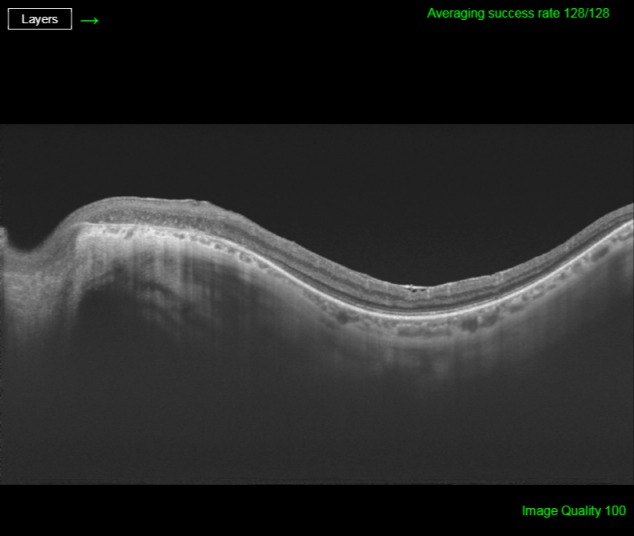
SS-OCT age of the left eye exhibits external retina damage with an increase in choroidal signal due to retinal pigment epithelium atrophy

His best corrected visual acuity (BCVA) was 20/32 (Snellen chart) in his right eye (OD) and 20/63 in his left one (OS). Anterior segment was unremarkable for both eyes (OU). Fundus evaluation showed pigment clumps along the retinal veins with variable chorioretinal atrophy extending from the disc up to the equator in both eyes (clinically more evident in OS). Fundus autofluorescence (FAF) imaging revealed hypo-autofluorescent areas corresponding to the atrophic patches over the posterior pole (Figure 1 (Fig. 1) and Figure 2 (Fig. 2)). SS-OCT showed a preserved foveal profile and great macular atrophy in OU, but serious involvement of the retinal outer layers in the left eye (Figure 3 (Fig. 3) and Figure 4 (Fig. 4)). In Angio-OCT, severe involvement of the ellipsoid, choriocapillar, and avascular layers could be appreciated in OS.

Electroretinography (ERG) showed diffuse response peaks in OU, with marked peripheral involvement, in addition to an increase in latency and a decreased amplitude in scotopic and photopic stimulation resulting from a generalized and advanced bilateral photoreceptor dysfunction accompanied by macular anomalies. In visual field (VF) 24.2, there was a higher altitude defect in OR and a temporary defect in OS. A clinical diagnosis of pseudo-paravenous atrophy secondary to Behcet’s disease OU was done.

## Discussion

PPRCA is a rare entity that is currently not well described. The cause of this condition may be unknown or idiopathic, although a dysgenetic, degenerative, hereditary etiology or even an inflammatory cause hav been hypothesized [5]. 

PPRCA primarily involves the RPE, with secondary atrophy of the underlying choroidal vasculature [6], [7], [8]. As the macula is often not affected, visual acuity keeps stable until advanced phases. Fundus appearance is quite characteristic, sometimes showing this paravenous atrophy and pigment accumulation. However, as other retinal cases can simulate PPRCA, we should support diagnosis with complementary tests. The use of a multimodal image can help us to differentiate this pathology from other similar pathologies as retinitis pigmentosa (RP) or acute zonal occult retinopathy (AZOR). 

Fundus fluorescein angiography (FFA) shows diffuse window defects with hyperfluorescence (consistent with RPE degeneration) and blockage of fluorescence (in areas with pigment clumping along the retinal vessels) can be showed. In the more severe early arterial phase, FFA aspect turns to extensive areas of choriocapillaris atrophy with prominently visible choroidal vessels along the major retinal veins adjacent to the disc. In the arteriovenous phase, delimited hyperfluorescence can be observed at the edge of the atrophic area, with hypofluorescence corresponding with the areas of pigment migration. 

Hypofluorescence in FAF shows the atrophy or disappearance of RPE because of a lipofuscin loss in RPE. Indeed, hypofluorescence detected by FAF corresponded to the areas of retinal thinning and atrophy detected using FA, IA, and OCT. In contrast, hyperfluorescence shows dysfunction of RPE that accumulates lipofuscin in RPE, suggesting the possibility of RPE atrophy in the future [9]. In the case that we present, paravenous hypoautofluorescence could be appreciated. 

SS-OCT scans normally reveal thinning of retinal layers with increased backscattering and disorganization of the RPE choriocapillaris complex. In many occasions, as we can see in our case, retinal and choroid slimming is such severe that scleral looks like very thick. Hyperreflective plaques with underlying shadowing corresponded to the pigment clumps observed clinically [10]. OCT-A study is not correctly defined yet and we do not find explanatory signs in our patient. 

Visual field shows peripheral scotomas corresponding to areas of chorioretinal atrophy. In advanced cases, the macula is also involved, so central visual field strategies as 10:2 VF should be used. 

Electrodiagnostic data are variable and nonspecific, ranging between normal and mildly affected, even markedly subnormal or a totally extinguished electroretinogram (ERG) [11]. This variation may signify that several conditions can present in this manner. In the case that we present, we can observe an alteration in photopic and scotopic response, more marked at periphery but with macular involvement too. Although some authors include PPCRA in the RP context based on clinical and electrophysiological state, PPRCA has a very slow progression compared to RP.

Diagnosis of pseudo-PPRCA can be achieved with a correct funduscopy suspect complemented with multimodal image study. 

Differential diagnoses include chorioretinal degeneration and inflammatory diseases that cause chorioretinal atrophy, including RP (pericentral, sector and typical), helicoid peripapillary chorioretinal atrophy, serpiginous choroidopathy, angioid streaks, cone dystrophy or degeneration, Stickler syndrome, gyrate atrophy choroideremia, Wagner’s dominant vitreoretinal degeneration, sarcoidosis, syphilis, acute retinal necrosis, cytomegalovirus retinitis, tuberculous disseminated choroiditis, onchocerciasis, toxoplasmosis, frosted branch angiitis, and various disorders that are termed pseudoretinitis pigmentosa. We have to keep in mind that this entity could present as an isolated phenomena or be associated to other ocular disorder as Behcet’s disease (as we found in our case), tuberculosis or syphilis [12]. 

## Conclusion

In summary, PPRCA is an uncommon disease, so exhaustive image studies must be performed for its correct definition and management. More cases would be needed to clarify the etiology of this entity and precise progression.

## Notes

### Competing interests

The authors declare that they have no competing interests.
